# Impact of Centrifugation Parameters on Platelet-Rich Plasma Injection for Patella Tendinopathy: A Systematic Review and Meta-Analysis

**DOI:** 10.7759/cureus.63341

**Published:** 2024-06-27

**Authors:** Edmund Jia Xi Zhang, Vincentius Edward Lie, Keng Lin Francis Wong

**Affiliations:** 1 Orthopedic Surgery, Yong Loo Lin School of Medicine, National University of Singapore, Singapore, SGP; 2 Orthopedic Surgery, Sengkang General Hospital, Singapore, SGP

**Keywords:** systematic review and meta analysis, patella tendon, meta-analysis, platelet-rich plasma, patella tendinopathy

## Abstract

Patellar tendinopathy (PT) is a chronic, degenerative form of tendinitis commonly affecting young, active individuals. Numerous nonsurgical treatments exist, of which platelet-rich plasma (PRP) is a frontrunner. However, heterogeneity among various PRP preparation techniques results in a large variation in treatment efficacy. This review and meta-analysis aims to investigate the effect of PRP centrifugation factors, specifically centrifuge speed and duration, on functional outcomes in patients with PT.

A systematic search of the literature was performed in April 2024 on Medline and Embase. Articles involving the use of PRP in the treatment of PT were included. The risk of bias was assessed using the Risk of Bias 2 (RoB 2; the Cochrane Collaboration, England, UK) and Risk of Bias in Non-randomised Studies of Intervention (ROBINS-I; the Cochrane Collaboration, England, UK) tools. Comparative meta-analysis between the different centrifugation speeds and the different centrifugation durations was performed on articles reporting Victorian Institute of Sports Assessment - Patellar tendon (VISA-P) and visual analogue scale (VAS) scores for PT.

Seventeen studies consisting of 360 participants were included in the analysis. The mean follow-up duration was 13.2 months (95% CI: 8.81 to 17.7). The mean VAS reduction was 3.85 (95% CI: -4.63 to -3.08; P < 0.01). VISA-P scores improved by 32.03 (95% CI: 24.29 to 39.78; P < 0.01). There were no significant differences between centrifuge speeds for VAS (P = 0.17) and VISA-P (P = 0.18) and between centrifuge durations for VAS (P = 0.25) and VISA-P (P = 0.27). Centrifuge speed and duration and number of centrifuge cycles did not show any significant differences in patient outcomes.

There were no significant differences observed in outcomes for the different preparations of PRP. There remains a need for further high-quality RCTs using standardized PRP preparations with long-term follow-up for the development of a consensus method of PRP preparation for the treatment of PT.

## Introduction and background

Patellar tendinopathy (PT) is a common disease process encountered in orthopedic sports medicine practice and is characterized clinically by anterior knee pain and tenderness over the inferior pole of the patella, with athletes forming a large proportion of PT patients [1]. PT is accentuated by repetitive, forceful, eccentric contractions of the extensor mechanism, with an estimated 14% of elite athletes and 8.5% of non-elite athletes being affected by PT at some point in their careers [2]. While physiotherapy has long been the mainstay in the management of PT, newer adjuncts such as extracorporeal shock wave therapy, therapeutic ultrasound, and injectables are showing promise. Andriolo et al. [1] found that injections of platelet-rich plasma (PRP) resulted in the best long-term patient outcomes for PT treatment. PRP has been shown to increase tendon cell proliferation, increase expression of anabolic genes and proteins, and reduce tendon inflammation [3].

Despite the increasing prominence of PRP usage in the treatment of PT, there remains no consensus on the method of PRP preparation. Factors such as centrifuge speed measured in rounds per minute (RPM), number of centrifuge cycles, and duration of centrifuges vary between preparation methods. Apart from methodological variance, contradictory results from similar preparation methods for PRP suggest that there is a lack of a clear paradigm regarding PRP preparation. Manfreda et al. and Vetrano et al. [4,5] found contradictory results at the same follow-up time despite sharing similar PRP preparation centrifuge speeds.

Hence, due to the variations in PRP preparation, we seek to compare the factors involved in the centrifugation. We hypothesize that there will be measurable differences among the various preparation factors in terms of patient outcomes and pain relief.

## Review

Methods

Search Strategy

This review was completed in accordance with the Preferred Reporting Items for Reviews and Meta-Analyses guidelines [6]. A search of the literature was performed using the Medline and Embase databases on April 1, 2024. Terms synonymous with “patellar tendinopathy” and “platelet-rich plasma” were used to construct the search strategy. References were managed with Endnote Reference Manager X9 (Clarivate Analytics, Philadelphia, PA, USA). Two separate independent authors (EZJX and EVL) performed the sieving of references. The literature search was limited to articles written in the English language. An example of the search strategy can be found within the supplementary material.

Study Selection

Studies involving patients treated with PRP for PT were included based on a predetermined inclusion criterion. The inclusion criteria were (I) studies involving skeletally mature patients undergoing PRP injections for PT, (II) studies presenting the pre- and post-treatment patient-reported outcome measure (PROM) scores, and (III) studies stating the centrifugation parameters. Quantitative methodology, including randomized controlled trials, retrospective and prospective cohort studies, and comparative studies, was included. Conference abstracts, reviews, case reports, editorials, opinions, and technical notes were excluded from this review. Additional grounds for exclusion were those of non-human or non-English publications. The final consensus on the articles used in the review was discussed between the two authors and a senior author (KLW).

Data Extraction

Data extracted from the included studies were (I) study information (author, title, sample size, country, and year of publication), (II) methodological variables (follow-up duration, number and volume of injections, PRP preparation type (kit), and the use of leukocyte poor PRP (LP-PRP) or leukocyte rich PRP (LR-PRP) injections), (III) patient characteristics (age, gender, BMI, and level of activity), and (IV) PROMs (Victorian Institute of Sports Assessment - Patellar tendon (VISA-P) and visual analogue scale (VAS)). Continuous data in the form of means and standard deviations were extracted from the articles. When data was presented as medians with ranges, we employed previously established formulae by Wan et al. [7] to convert the data to means with standard deviations.

Risk of Bias

The risk of bias in non-randomized trials was assessed with the Risk of Bias in Non-randomised Studies of Intervention (ROBINS-I; the Cochrane Collaboration, England, UK) tool [8], and the risk of bias in randomized trials was assessed with the Risk of Bias 2 (RoB 2; the Cochrane Collaboration, England, UK) tool [9]. The ROBINS-I and RoB 2 tools are specialized tools structured into fixed sets of domains of bias, ultimately categorizing studies into various levels of risk of bias: low, moderate, serious, and critical. These tools provide a structured and comprehensive approach to assessing interventions through signaling questions to determine overall risk [8].

Outcomes of Interest

This study primarily aimed to evaluate the factors affecting the reduction in pain and improvement of patella function after the administration of PRP. Primary outcomes of interest were those of the VAS for pain as well as the hundred-point VISA-P questionnaire for patella function. The VISA-P is a quantitative measure of activity and sporting limitations, as well as a surrogate marker of PT symptom severity. Pre-injection and post-injection scores were compared.

Factors Affecting PRP Preparation

We chose to evaluate the following factors as variables affecting the preparation of PRP injections: (I) spin speed - the speed at which the samples were centrifuged, characterized as high (>2000 RPM or >500 G) and low centrifuge speed (<2000 RPM or <500 G). (II) Spin time - the duration at which the centrifuge of samples was taken, characterized by long (>10 minutes) and short (<10 minutes) spin times. (III) Spin cycles - PRP injection preparation by single or double spin. Double spin refers to centrifuging the samples twice, at different spin speeds and spin times, while single spin is centrifuging the samples once. Double-spin kits include the Gravitational Platelet Concentration System® (GPS) III (Biomet Inc., Warsaw, Indiana, USA) PRP kit, while single-spin kits include the GPS II Platelet Concentration System (Biomet Inc., Warsaw, Indiana, USA), Arthrex (Angel) (Naples, Florida, USA), Regen Lab A-PRP Kit (Regenlab, Switzerland), Arthrex (ACP) (Naples, Florida, USA), and Arthrex Nederland B.V. 15-ml ACP double syringe (The Netherlands) and MyCells Autologous Platelet Preparation System (Ramat Hasharon, Tel Aviv, Israel). Studies that had unspecified PRP preparation systems were classified according to their method of preparation [10-13].

Statistical Analysis and Quality Assessment 

Statistical analysis was performed using Review Manager Web (The Cochrane Collaboration, Copenhagen, Denmark). Continuous data, in the form of pre- and post-treatment PROM scores, was pooled using the inverse variance model to generate weighted mean differences (WMD) as a surrogate measure of treatment effect. Analysis using the random effects model was undertaken regardless of study heterogeneity, as represented by the I2 statistic. Demographic data was analyzed in a single-arm fashion to obtain raw weighted means using the inverse-variance model with random effects. This analysis was performed using the metamean function in RStudio version 1.3.1093 (RStudio, Boston, Massachusetts).

Subgroup analyses were conducted for the number of PRP injections and the type of preparation method used for the injections as a means of further exploring study heterogeneity.

Results

Summary of Included Articles

**Figure 1:**
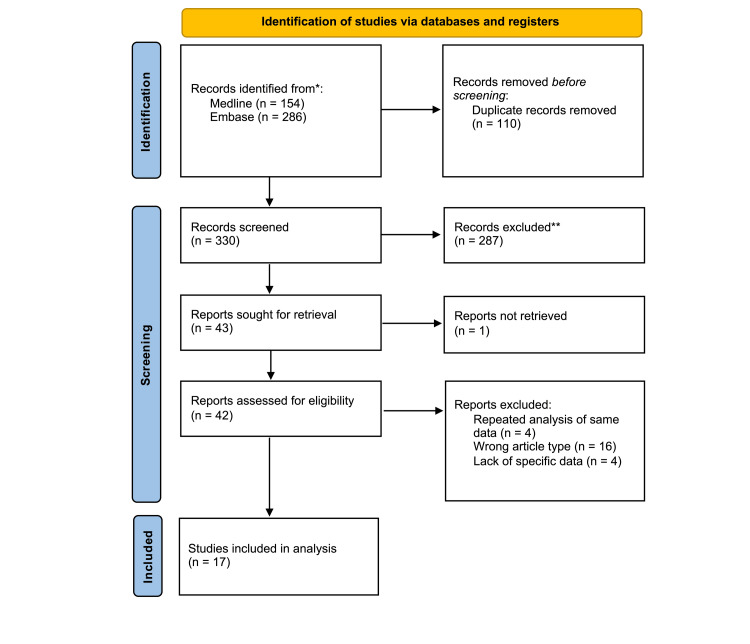
PRISMA diagram of the included studies PRISMA: Preferred Reporting Items for Systematic reviews and Meta-Analyses

A systematic search of the literature using our search strategy yielded 330 references, of which 287 were excluded based on the study title and abstract. Forty-three articles underwent full-text review, and 17 articles concerning the use of PRP for PT met the eligibility criteria and were subsequently included in the meta-analysis (Figure 1). In total, 360 patients underwent PRP injections for PT. Overall, seven articles originated from Italy [2,4,5,12-15]; two each from the USA [11,16], France [17,18], and the Netherlands [19,20]; and one each from Egypt [21], Turkey [10], and Spain [22], as well as a multicenter study that involved hospitals in Italy, Norway, and the USA [23]. A summary of the study characteristics of the included articles can be found in Table 1.

**Table 1:** Summary of included studies RNR: retrospective nonrandomized study, PNR: prospective nonrandomized study, RCT: randomized controlled trial, BMI: body mass index, VISA-P: Victoria Institute of Sport Assessment - Patella tendon, VAS: visual analogue scale [2,4,5,10-23]

Author	Year	Study design	N	Age (years)	Male (%)	BMI (kg/m2)	Preparation system	Speed	Duration	Outcomes
Abate et al. [2]	2018	RNR	18	38.6	38.9	25.0	Regen Lab	High	Short	VISA-P, VAS
Manfreda et al. [4]	2019	PNR	17	30.6	70.6	24.3	Biomet GPS	High	Long	VISA-P, VAS
Vetrano et al. [5]	2013	PNR	23	26.9	86.9	-	MyCells	Low	Long	VAS
Unlu et al. [10]	2017	RNR	37	33.2	37.8	-	-	Low	Short	VAS
Dallaudiere et al. [11]	2014	RNR	41	-	-	-	-	High	Long	VAS
Filardo et al. [12]	2010	PNR	15	28.8	100.0	-	-	Low	Long	VAS
Filardo et al. [13]	2013	PNR	43	30.6	97.7	24.7	-	High	Short	VISA-P, VAS
Volpi et al. [14]	2007	RNR	7	27	-	-	Biomet GPS	High	Long	VISA-P
Crescibene et al. [15]	2015	PNR	7	-	-	-	Regen Lab	High	Short	VISA-P, VAS
Dragoo et al. [16]	2014	RCT	8	28	89.0	-	Biomet GPS	High	Long	VISA-P, VAS
Charousset et al. [17]	2014	PNR	28	27.3	-	21.6	Arthrex (ACP)	Low	Short	VISA-P, VAS
Zayni et al. [18]	2015	PNR	30	24.6	87.5	-	Arthrex (ACP)	High	Short	VISA-P, VAS
Ark et al. [19]	2013	PNR	5	27	40.0	25.7	Arthrex (ACP)	High	Short	VISA-P
Gosens et al. [20]	2012	PNR	22	32.2	63.3	-	Biomet GPS	High	Long	VISA-P, VAS
Abdelbary et al. [21]	2018	PNR	10	35.5	-	-	Arthrex (ACP)	Low	Long	VAS
Soler et al. [22]	2023	PNR	10	-	-	-	-	Low	Short	VISA-P, VAS
Scott et al. [23]	2019	RCT	19	32	90.0	24.0	Arthrex (Angel)	High	-	VISA-P

Patient Demographics

A total of 360 patients with a mean age of 29.6 (95% CI: 27.8 to 31.5) underwent PRP treatment for PT. The mean follow-up duration was 13.2 months (95% CI: 8.81 to 17.7 months). In total, 237 patients received PRP prepared using a high centrifuge speed, while 123 received PRP that was centrifuged with a low spin speed. Furthermore, a total of 161 patients received PRP injections with long centrifuge times, while 188 patients received PRP injections with short centrifuge times. A total of 58 patients received double-spin PRP.

Risk of Bias

**Figure 2:**
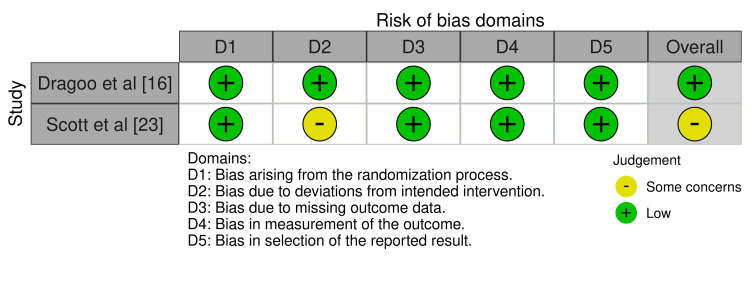
RoB 2 traffic light plot RoB 2: Risk of Bias 2 [16, 23]

**Figure 3:**
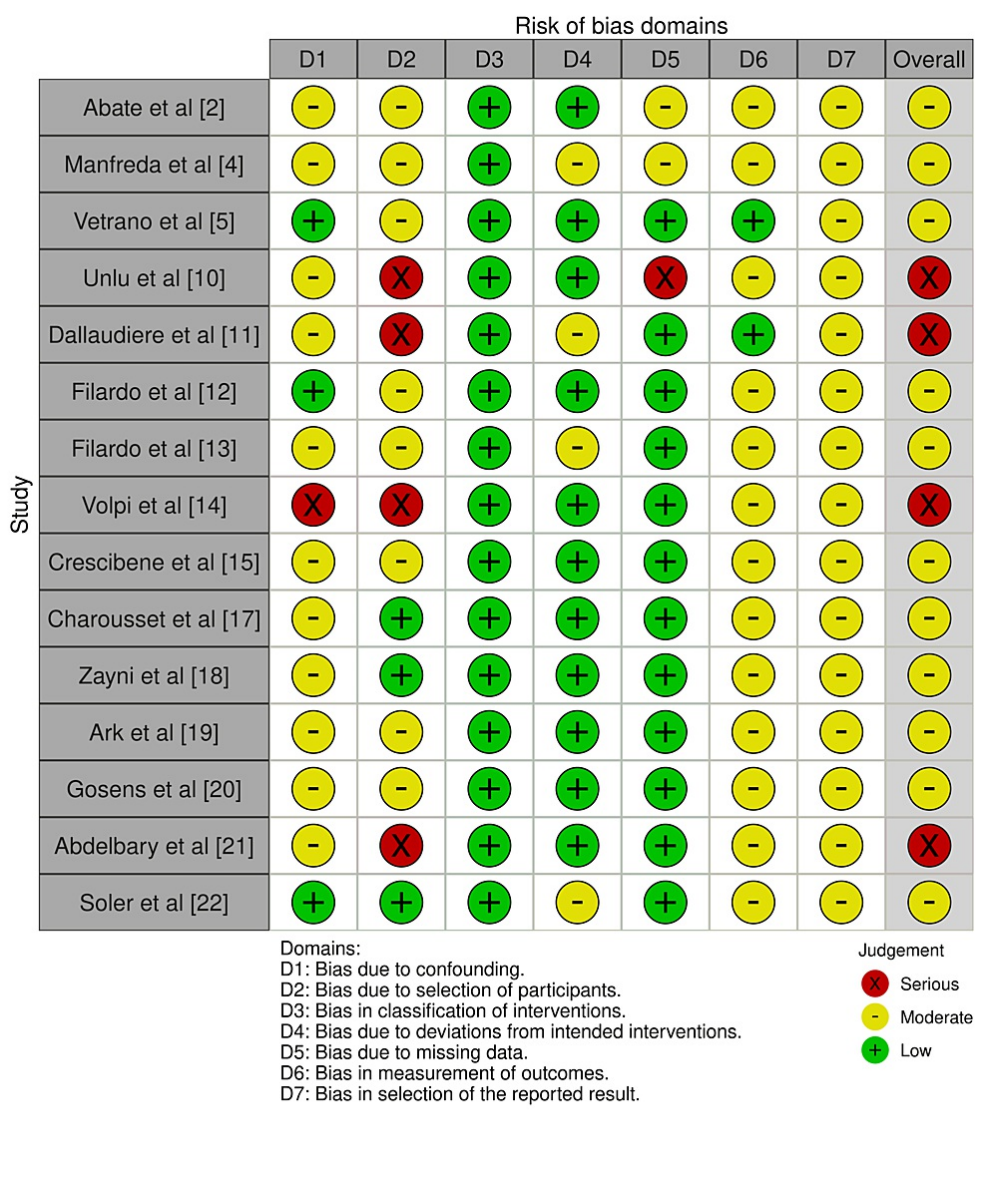
ROBINS-I traffic light plot ROBINS-I: Risk of Bias in Non-randomised Studies of Intervention [2,4,5,10-15,17-22]

The majority of the non-randomized studies analyzed exhibited a moderate risk of bias. While they are more susceptible to biases as compared to randomized trials, these risk levels do not invalidate their findings but suggest cautious interpretation. The assessment of biases for non-randomized trials and randomized trials is shown below (Figures 2-3).

Summary of Results

The VAS score was assessed in 13 studies consisting of 251 patients, with an average reduction in VAS of 3.85 (95% CI: -4.63 to -3.08; P < 0.01). Similarly, the VISA-P score was assessed in 13 studies comprising 261 patients, and an improvement in the VISA-P score of 32.03 (95% CI: 24.29 to 39.78; P < 0.01) was seen post-treatment following PRP injections in the patella. There were no significant differences between centrifuge speeds for VAS (P = 0.17) and VISA-P (P = 0.18) or between centrifuge durations for VAS (P = 0.25) and VISA-P (P = 0.27).

Centrifuge Speed

**Figure 4:**
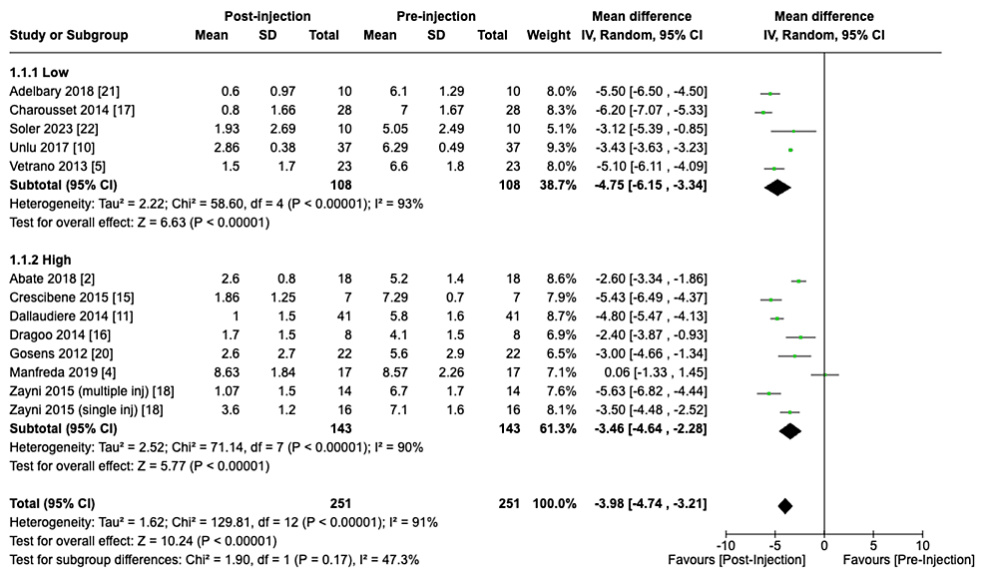
Effect of centrifuge speed on VAS scores VAS: visual analogue scale [2,4,5,10,11,15-18,20-22]

A subgroup analysis was performed comparing different centrifuge speeds utilized in PRP preparation on VAS and VISA-P post-treatment score improvements. Pooled analysis revealed a weighted mean VAS score reduction of 4.75 (95% CI: -6.15 to -3.34; P < 0.01) and 3.46 (95% CI: -4.64 to -2.28; P < 0.01) in the low and high centrifuge speed groups, respectively. However, the difference in VAS score reduction between low and high centrifuge speed subgroups was not statistically significant (P = 0.17) (Figure 4).

**Figure 5:**
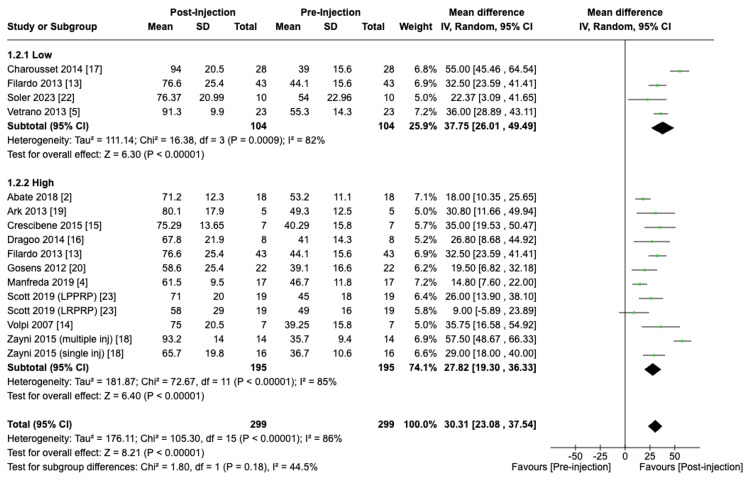
Effect of centrifuge speed on VISA-P scores VISA-P: Victorian Institute of Sports Assessment - Patellar tendon [2,4,5,13-20,22,23]

Patients in the low centrifuge speed group exhibited greater improvement in VISA-P scores (WMD = 37.75; 95% CI: 26.01 to 49.49; P < 0.01) as compared to the high spin speed group (WMD = 27.82; 95% CI: 19.30 to 36.33; P < 0.01); however, this difference in VISA-P score improvement was not statistically significant (P = 0.18) when comparing between the two groups (Figure 5).

Duration of Centrifuge

**Figure 6:**
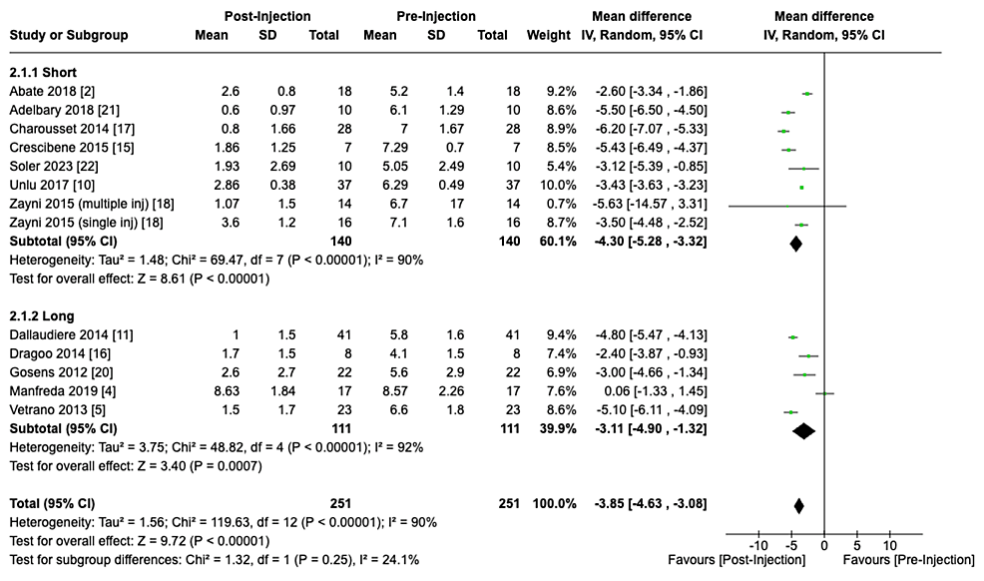
Effect of Centrifuge Duration on VAS Scores [2, 4, 5, 10, 11, 15-18, 20-22]

VAS scores showed a reduction of 4.30 (95% CI: -5.28 to -3.32; P < 0.01) and 3.11 (95% CI: -4.90 to -1.32; P < 0.01) with the utilization of short and long centrifuge durations, respectively. However, no significant differences were observed when comparing the differences in VAS score reduction between the short and long-spin duration subgroups (P = 0.25) (Figure 6).

**Figure 7:**
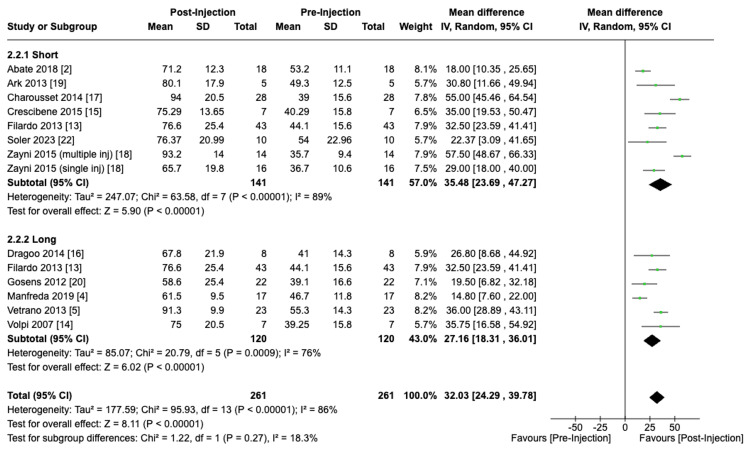
Effect of centrifuge duration on VISA-P scores VISA-P: Victorian Institute of Sports Assessment - Patellar tendon [2,4,5,13-20,22]

VISA-P scores showed an improvement of 35.48 (95% CI: 23.69 to 47.27; P < 0.01) and 27.16 (95% CI: 18.31 to 36.01; P < 0.01) with the utilization of short and long spin durations, respectively. Similarly, no significant differences were found in terms of the degree of VISA-P score improvement when comparing both centrifuge duration subgroups (P = 0.27) (Figure 7).

Number of Centrifuge Cycles

A VISA-P score was reported by one study that utilized double-spin PRP preparation. A sensitivity analysis was conducted to exclude this study from the overall analysis (WMD = 30.25; 95% CI: 21.64 to 38.35; P < 0.01), revealing a VISA-P score improvement of 30.02 (95% CI: 20.53 to 39.51; P < 0.01) post-sensitivity analysis. Additionally, the effect of the number of centrifuge spin cycles on VAS score improvement could not be evaluated due to a lack of data on the utilization of a double spin method.

Discussion

This study is the first to assess the impact of PRP preparation variables on clinical outcomes in patients with PT. Our study demonstrated that individual preparation variables do not lead to better clinical outcomes in terms of function and pain relief.

PT is a debilitating condition, especially in patients with high functional demands, with chronic, untreated PT often resulting in significant functional loss [23]. Additionally, repetitive loading of a patellar tendon with pre-existing pathology may engender changes in the biomechanical properties of the tendon, accentuating the risk of tendon rupture [24]. In such cases, surgical treatment is often required, with extensor mechanism failure being a definitive indication for surgical intervention [25]. Hence, the current paradigm in the treatment of PT centers around regenerative therapy aimed at correcting underlying pathologies within the tendon to reduce the risk of progression to rupture. Andriolo et al. [1] identified PRP therapy as the most effective conservative treatment of PT. PRP has been shown to increase tendon cell proliferation, increase the expression of anabolic genes and proteins, and reduce tendon inflammation [3]. However, the method of PRP preparation remains largely heterogenous, and there is no consensus regarding the optimal preparation and centrifugation conditions. Numerous factors, such as the centrifuge speed and duration, as well as the number of centrifuge cycles, can affect the platelet yield and purity of the PRP, directly affecting the concentration of growth factors and thereby influencing the quality of the PRP, which could potentially give rise to considerable heterogeneity in patient outcomes.

Administration of PRP for PT displayed an overall reduction in pain scores as well as improvements in functional scores. However, attempts at investigating the effect of individual preparation variables on clinical outcomes did not display convincing trends of evidence. Patients receiving PRP prepared via a low centrifuge speed were observed to have greater reductions in VAS score and improvements in VISA-P score; however, these inter-variable differences were not statistically significant. Therefore, limited conclusions can be drawn, and the improvements conferred by PRP prepared at a lower centrifuge speed have limited interpretability that is germane to clinical significance. Hence, no recommendation can be made for a suggested centrifuge speed at this point in time. Similarly, when evaluating centrifuge duration as a factor for PRP preparation, marginally greater VAS reduction and VISA-P improvements were observed in the short centrifuge duration group. However, the difference in both score improvements between long- and short-duration centrifuge cycles was neither statistically significant nor reached minimal clinically important difference values [26], potentially suggesting that centrifuge duration may not be an important factor in PRP preparation.

Separation of cellular constituents within the autologous blood derived for PRP treatment depends on several factors, such as the relative centrifugal force (RCF), which can be adjusted to sediment certain particles more quickly than others [27]. In the preparation of PRP, erythrocytes sediment at the base, leaving the supernatant that contains plasma, platelets, and white blood cells available for separation from the erythrocytes. The autologous platelet concentrate obtained by centrifugation thus induces various growth factors that promote the regeneration of the tendon. Platelet yield is determined by the sedimentation rate governed by Stokes’ law, with the RCF influencing the separation of platelets from erythrocytes [28]. Centrifuging PRP with a greater centrifugal force increases the sedimentation rate and the separation of platelets from erythrocytes, thereby enhancing platelet capture efficiency. However, our analysis showed that higher centrifuge speed (>2000 RPM or >500 G) does not positively influence the post-injection outcomes, as seen in the non-significant differences with the improvements in VAS and VISA-P scores when compared to PRP centrifuged at a lower speed. This increase in platelet capture efficiency could be offset by a potential compromise on platelet preservation and integrity when it was spun at a high centrifuge speed [29], thereby affecting the bioactivity of platelets and stimulating the proliferation of several cell types for tissue regeneration and healing. Nonetheless, this suggests that centrifuge speed is not a parameter that defines the composition of PRP.

Conversely, we noted that the centrifuge duration of PRP contributes little to the enhancement of post-injection outcomes, wherein the difference between the use of a short centrifuge duration (<10 m) and a long centrifuge duration (>10 m) displayed insignificant improvements in pain and functionality. When centrifuging a PRP sample, the sedimentation of erythrocytes allows for the collection of platelets and plasma from the upper layer due to their different sedimentation rates. Once most of the erythrocytes are sedimented, further increasing the centrifuge duration would not enhance the separation of erythrocytes from the platelets. As such, since the platelet yield does not increase appreciably after a certain threshold, these findings suggest that centrifuging the PRP sample for differing durations minimally contributes to the quality of the PRP obtained.

Limitations

Firstly, we were limited by the heterogeneity within the data of the included studies, with various follow-up times, center practices, and individual variations in the techniques of the surgeons contributing to the wide confidence intervals observed. Furthermore, as with any meta-analysis, there is an inherent degree of statistical heterogeneity, as measured by the I2 statistic. However, where possible, we attempted to explore heterogeneity through subgroup and sensitivity analyses, where appropriate. Finally, this review is limited in terms of interpretability for the long-term outcomes of PRP usage in PT.

## Conclusions

Despite the increasing usage of PRP as a conservative treatment for PT, PRP is limited by the heterogeneity of its preparation methods and the wide variation in its centrifugation protocol, yielding various platelet concentrations. There is a need for further high-quality RCTs using standardized PRP preparations with long-term follow-up for the development of a consensus method of PRP preparation for the treatment of PT. In addition, future studies may explore the effects of different centrifugation parameters beyond pain, such as on various functional outcomes and quality of life. This study has analyzed the effects of centrifuge speed and duration, as well as the number of centrifuge cycles, on the quality of PRP and concluded that centrifuge speed and duration may have no significance in the quality of PRP produced.

## Appendices

**Table 2:** Search strategy

Medline	
1	exp Tendon Injuries/
2	exp Tendinopathy/
3	tendin*.tw. or tendon*.tw.
4	(Tendon adj3 injur*).tw.
5	or/1‐4
6	exp Patellar Ligament/
7	patellar.tw.
8	6 or 7
9	5 and 8
10	exp Platelet‐Derived Growth Factor/
11	(platelet‐derived growth factors or PDGF).tw.
12	exp Blood Platelets/
13	(platelet rich plasma or platelet‐rich plasma or PRP or platelet gel*).tw.
14	exp Platelet Activation/
15	(platelet* adj activat*).tw.
16	or/10-15
17	9 AND 16
Embase	
1	‘tendon injury’
2	tendinitis/exp
3	(tendon* or tendin*):ti,ab
4	(Tendon next/3 injur*):ti,ab
5	#1 or #2 or #3 or #4
6	‘patellar ligament’/exp
7	patellar*:ti,ab
8	#6 or #7
9	#5 AND #8
10	‘Platelet Derived Growth Factor’/exp
11	(‘platelet‐derived growth factors’ or ‘PDGF’):ti,ab
12	Thrombocyte/exp
13	‘Thrombocyte Rich Plasma’/exp
14	(‘platelet rich plasma’ or ‘platelet‐rich plasma’ or ‘PRP’ or ‘platelet gel*’):ti,ab
15	‘Thrombocyte Activation’/exp
16	(platelet* next/1 activat*):ti,ab
17	#10 or #11 or #12 or #13 or #14 or #15 or #16
18	#9 AND #17
